# Recycled leather–epoxy hybrid composites exhibiting novel fracture behavior identified through multimodal characterization

**DOI:** 10.1038/s41598-025-32862-6

**Published:** 2025-12-22

**Authors:** Sampath Suranjan Salins, Sawan Shetty, Deepak Doreswamy, Thamme Gowda, H. K. Sachidananda

**Affiliations:** 1 School of Engineering and IT, Manipal Academy of Higher Education, Dubai Campus, Dubai, UAE; 2 Manipal Institute of Technology, Manipal Academy of Higher Education, Manipal, India; 3 Department of Mechanical Engineering, Vidyavardhaka College of Engineering, Mysuru, Karnataka India

**Keywords:** Leather-epoxy composites, Bio-based hybrid composites, Flexural properties, Fracture behavior, Microstructure, Sustainable materials, Engineering, Materials science

## Abstract

This study investigates the mechanical behavior and fracture mechanisms of recycled leather-epoxy composites fabricated via resin infusion and evaluated under quasi-static three-point bending. The work employs industrial post-consumer leather waste, mechanically processed into fibrous form, as a sustainable reinforcement alternative to conventional natural fibers. At a fiber volume fraction of approximately 0.3, the composites achieved a flexural strength of 100.8 ± 1.92 MPa and a modulus of 18.64 ± 0.38 GPa, showing favorable performance relative to reported jute-epoxy and flax-epoxy systems under comparable testing conditions. The bilinear viscoelastic-softening model captured the composite stress–strain response and an estimated critical energy release rate (Gc ≈ 8.89 × 10^−3^ J/m^2^) reflected the onset of progressive delamination. Micro-voids and resin-deficient zones associated with collagen-based fiber morphology acted as stress concentrators that shaped local failure events. This exploratory study advances understanding of micromechanical toughening in bio-derived composites and indicates that processed post-consumer leather fibers exhibit promising flexural behavior and damage tolerance, suggesting potential for semi-structural or moderately loaded lightweight applications pending further durability and service-level validation

## Introduction

The global emphasis on sustainable materials has intensified interest in incorporating agro-industrial waste into composite systems. Among various waste-derived reinforcements, leather waste remains underutilized despite its high collagen content, fibrous morphology, and inherent toughness. Discarded leather from upholstery and footwear industries poses significant environmental challenges due to its slow degradation and limited recycling pathways. Converting this waste into functional reinforcement for polymer composites offers a dual benefit: mitigating environmental burden while generating high-performance, cost-effective materials for semi-structural applications.

Leather-epoxy composites integrate processed leather fibers within an epoxy matrix, combining the inherent toughness of collagen-based fibers with the stiffness of thermoset polymers. This composite offers enhanced mechanical properties^1,2^, improved resistance to environmental factors^3,4^, and extended longevity, making it suitable for various industrial and commercial applications. Leather epoxy composites are widely used in the automotive and aerospace industries for lightweight yet durable interior components, in fashion and accessories for reinforced leather goods, and in furniture manufacturing for high-end, wear-resistant upholstery. Ullisses et al.^5^ have studied effect of graphene oxide in case of natural fibers owing to lower cost and density and ease of processing. They used these natural fibers for diversified applications in case of multilayered ballistic armor. They concluded that these natural composites effectively dissipate the projectile impact energy. Ullisses et al.^6^ have studied the mechanical properties of composites with graphene oxide in case of epoxy matrix reinforcement to investigate the influence of graphene oxide on the tensile properties. They concluded that the increase in tensile strength, young’s modulus and toughness was observed in these composites with graphene oxide. Flavia et al.^7^ have studied statistically guide development and optimization of graphene-nanoplatelets reinforced epoxy composites using a box-behnken design. They prepared these composites by varying the proportions of graphene-nanoplatelets and comprehensive characterization and tensile testing has been performed. They concluded that these statistically optimized composites can perform better as compared to neat epoxy composites. Manjunathan et al.^8^ has studied impact of fiber composition, sequence and stacking pattern in case of hybrid composites encompassing varied stacking sequence by using fiber of jute, bamboo and glass using hand-layup process. They positioned the jute of bamboo fiber in perpendicular direction with respect to adjacent piles. They concluded that these arrangements increase strain and toughness. Ertan et al.^9^ has studied mechanical properties of carbon fiber reinforced epoxy composite filled with graphene and silicon carbide at different weight ratios. These composites were tested for tensile strength, compression and three-point bending. They concluded that the elastic modulus was higher in these composites as compared to unfilled composites. Also, the results were not having positive effects on compression strength, whereas the results are positive for three point bending strength. Jiajia et al.^10^ has investigated the effect of SiC nanoparticle on microstructure and mechanical properties of graphene nanosheet reinforced aluminum composites using a ball milling process. This ball milled AI flakes effectively improved the dispersion efficiency and reduced the structural damage of graphene nanosheet reinforced aluminum composites. They concluded that composite material showed significant improvement in young’s modulus and tensile strength.

Ravindra and Nilesh^11^ have investigated waste leather-reinforced epoxy composites by fabricating sheets with varying leather content and thicknesses of 2.5 mm, 5 mm, and 7.5 mm using the surface response methodology. The samples underwent tensile, flexural, impact, and chemical resistance testing. The findings revealed that the composite demonstrated exceptional mechanical properties, with SEM analysis confirming strong packing and adhesion within the composite matrix. Additionally, the composites exhibited excellent chemical resistance. Laksoman and veena^12^ have examined leather composites using two different types of leather waste, analyzing their effects on mechanical properties, including hardness, tensile strength, and thermal stability. The researchers compared these composites with unfilled leather composites and found that the leather-reinforced composites demonstrated superior strength. Iva et al.^13^ have explored industrial leather by developing composites with epoxy and high-density polyethylene. The findings suggested that incorporating leather enhanced the average specific compression toughness of epoxy by 29%. Fracture surface analysis revealed that the presence of leather microparticles facilitated a transition in failure mode from brittle to ductile. Shubham et al.^14^ have explored the use of leather waste to develop poly(ethylene–vinyl-acetate) (EVA)-based polymer composites for applications in flooring, structural components, footwear, and transportation. The specimens were evaluated for compressive and tensile strength, abrasion resistance, density, tear resistance, hardness, adhesion strength, and compression properties. SEM and EDAX analyses confirmed excellent uniformity, compatibility, stability, and strong bonding of leather fibers within the composite matrix. Macaulay et al.^15^ examined the impact of varying processing temperatures on the thermal and mechanical properties of uncoated and coated epoxy leather composites. The findings indicated that coated epoxy leather exhibited superior mechanical properties compared to uncoated composites, with these results further validated through field emission scanning electron microscopy (FESEM).

Earlier work by Ravichandran and Natchimuthu^16^ demonstrated the feasibility of incorporating leather waste into polymer composites, enabling applications in construction, automotive, and other industrial sectors. Their findings laid the groundwork for the safe and effective processing of leather waste within composite systems. Building on this foundation, recent studies have shifted focus toward sustainability, interfacial performance, and detailed microstructural behavior. For instance, Martínez-Hernández et al.^17^ reviewed the use of agro-industrial waste as sustainable reinforcement in bio-based polymer composites, emphasizing the contribution of such materials to circular economy objectives and eco-friendly material design. These developments highlight leather waste’s evolution from a passive filler to active reinforcement, capable of improving both mechanical performance and structural reliability in thermosetting composite systems.

Jun et al.^18^ investigated composites produced by incorporating leather fibers into nitrile rubber. The blend was milled, vulcanized, and subsequently processed to obtain a stabilized leather–rubber composite designed for sealing and related functional applications. They concluded that the resulting material demonstrates adequate hardness and thermal stability, making it a viable option for use in sealing components. Barrera et al.^19^ have investigated the mechanical and thermal properties of rubber composites developed using leather waste to promote sustainability. They concluded that these composites hold potential for use in the fashion industry, offering opportunities to create innovative designs that incorporate waste and residues as part of a natural and eco-friendly design approach. Basak et al.^20^ have investigated naturally based flexural composites formulated using chemically treated natural rubber. They examined both physical and performance properties, including abrasion resistance, tensile strength, and tear strength. Their study concluded with a comparative analysis of the morphology between the developed flexural composites and natural leather. Muhammad et al.^21^ have investigated upcycling leather waste by incorporating it in thermoplastic polyurethane, they prepared these specimens with different tanning methods and with different particle size distribution and studied the morphological, thermal and mechanical properties. They concluded that these composites have enhanced mechanical properties and abrasion resistance by average particle size of the leather waste. Rui et al.^22^ has studied unidirectional carbon/glass hybrid reinforced polymer composite rods using the pultrusion method. They studied the effect of fiber hybridization types on the mechanical properties considering three-point bending, shear and tensile strength. They concluded that the tensile strength of carbon fiber contributed to higher mechanical strength.

From the above literature it is observed that researchers have extensively investigated the use of leather waste in various composite systems, particularly in rubber and polymer matrices, with the aim of enhancing sustainability and material efficiency. These studies have demonstrated that leather-based fillers can improve mechanical performance, such as tensile strength, abrasion resistance, and thermal stability, while offering an environmentally friendly alternative to traditional reinforcement materials^23^. Applications of such composites have been explored in sectors like construction, automotive, and fashion, highlighting the potential of leather waste in creating functional and aesthetic materials.

Despite their promise as sustainable and flexible reinforcements, leather-epoxy composites present several challenges that warrant consideration. A key limitation lies in the inherent variability in leather fiber morphology and chemistry, which can lead to inconsistent interfacial bonding with the epoxy matrix. Unlike engineered fibers, leather fibers often contain natural oils, collagen irregularities, and non-uniform cross-sections, which may hinder effective wetting and adhesion. Additionally, manual processing methods, such as hand lay-up or uncontrolled resin infusion, may result in poor fiber dispersion, void formation, or resin-starved regions, ultimately affecting mechanical uniformity and reproducibility. The heterogeneous nature of leather waste, especially when sourced from industrial by-products, can also introduce batch-to-batch variability in mechanical performance. These drawbacks highlight the need for surface modification strategies, improved processing techniques, and more systematic design methodologies to fully harness the potential of leather-based composites in structural applications.

Building on these findings, the present study examines the development of leather–epoxy composites by incorporating mechanically processed leather fibers into an epoxy matrix. The work focuses on evaluating flexural performance under three-point bending and analyzing fracture behavior using micromechanical and fracture-mechanics frameworks. The investigation also explores the potential suitability of leather–epoxy composites for moderately loaded applications where mechanical reliability and sustainable material sourcing are desirable, including interior components, lightweight panels, and environmentally conscious product. While studies have demonstrated the use of leather waste in rubber, thermoplastics, and polymer matrices, most investigations have prioritized chemical compatibility or bulk mechanical characterization without systematic insights into the fracture mechanics, failure pathways, and micromechanical interactions of leather-reinforced epoxy systems. Moreover, few works have employed multimodal characterization combining three-point bending, optical microscopy, SEM imaging, and micromechanical modeling to holistically understand the structural behavior of such composites.

From an application standpoint, leather epoxy composites hold significant potential in sectors where lightweight materials with moderate mechanical strength and enhanced sustainability are desirable. Due to the fibrous structure and natural resilience of processed leather waste, such composites can serve as viable alternatives in non-structural and semi-structural components within the automotive interior, footwear, consumer goods, and furniture industries. Their use not only contributes to material circularity by valorizing post-consumer leather waste but also aligns with the growing demand for eco-efficient composite solutions. Therefore, understanding the mechanical performance and failure mechanisms of leather epoxy composites is essential to evaluate their suitability for such emerging applications. This study aims to bridge this knowledge gap by systematically characterizing the composite’s structural integrity, reinforcing behavior, and fracture response.

The introduction of the current study can be further enriched by drawing upon recent advancements in the development of sustainable, bio-based composites. For instance, the study by Vinod et al.^24^ on the development of a fully bio-based sport utility component using jute/hemp-reinforced bio-epoxy composites demonstrates how stacking sequence significantly influences fatigue performance, thermo-mechanical properties, vibrational response, and viscoelastic behavior. Similarly, the work of Gokul kumar et al.^25^ investigates hybrid composites fabricated from flax, vetiver, and Luffa cylindrica, highlighting their potential for both acoustic insulation and structural reinforcement. These contributions underscore the growing relevance of bio-composite systems in functional and structural applications, offering valuable comparative insights for the present investigation on leather epoxy composites. Situating this study within such a framework not only emphasizes its relevance to the field of sustainable materials engineering but also positions leather waste as a viable reinforcement alternative aligned with circular economy principles.

Building on recent advancements in bio-based composite development, the present study aims to explore the viability of incorporating waste-derived leather fibers into epoxy matrices to develop sustainable hybrid composites. While prior research has established the utility of natural fibers such as jute, hemp, flax, and Luffa cylindrica for enhancing mechanical, thermal, and acoustic properties, limited attention has been given to valorizing leather waste in composite systems. In this context, the present study aims to fill three key gaps in the literature:

1. To assess the flexural performance of leather-epoxy composites fabricated via controlled resin infusion and subjected to standard bending protocols;
2. To elucidate failure mechanisms and toughening behavior through multi-scale microscopy and predictive modeling;
3. To evaluate the potential of processed leather fibers as sustainable, structurally viable reinforcements comparable to traditional biofibers such as jute or flax.

To the best of our knowledge, this is the first study to present a multimodal correlation of three-point bending performance, optical microscopy, scanning electron microscopy (SEM), and micromechanical modeling in leather-epoxy composites. This integrated approach enables a comprehensive understanding of fracture pathways, fiber-matrix interactions, and energy dissipation mechanisms, thereby extending the current scope of leather-based composite research beyond bulk mechanical testing.

The toughening mechanisms identified here such as fiber pull-out, debonding, and crack deflection are consistent with those reported for jute, flax, and kenaf systems, although the collagen-based leather fibers generate more diffuse interfacial debonding and shear-driven energy dissipation compared with the more anisotropic lignocellulosic fibers.

## Methodology

### Materials

**Fig. 1:**
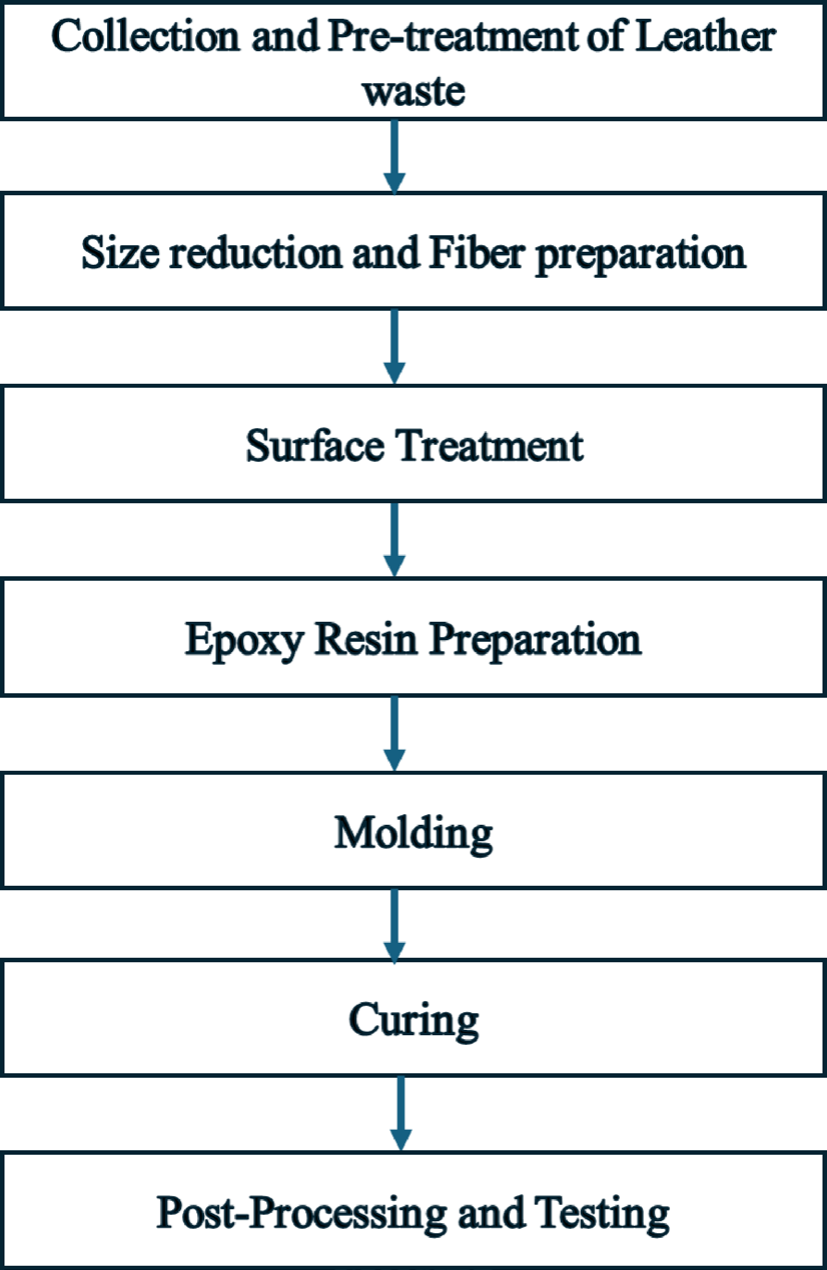
Step by step process for preparing leather epoxy composites.

Post-consumer leather waste was sourced from upholstery and footwear industries. These leather scraps, devoid of any biological or human/animal testing, were used solely for laboratory experimentation, adhering to institutional policies on chemical handling and sustainability. Therefore, ethical approval was not required. Araldite LY 556 epoxy resin and Aradur HY 951 hardener (Huntsman, Germany) were used as the matrix system in a 100:10 weight ratio. All reagents were used as received. Moisture-free conditions were maintained using sealed desiccator storage (Refer Fig. 1).

### Preparation of leather reinforcement

**Fig. 2:**
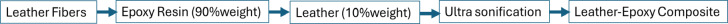
Schematic representation of the sequential preparation of leather fibers for composite fabrication, including cleaning to remove contaminants, mechanical shredding, particle size uniformity through sieving, and dry storage to prevent moisture-induced degradation before mixing with epoxy resin.

Leather waste was initially cleaned in distilled water and dried in a Memmert UN30 laboratory oven at 60 °C for 24 h to remove residual moisture. The dried material was mechanically shredded using a REMI RQ-122 high-speed stirrer to produce short fibers (3–5 mm length, 0.5–1 mm width). For powder composite trials, the shredded leather was pulverized and sieved through a 60-mesh (250 µm) screen using a Fritsch Analysette 3 Spartan Vibratory Sieve Shaker. However, only fiber-reinforced specimens were evaluated in this study. All processed fibers were stored in airtight polyethylene containers with silica gel to prevent moisture absorption (Refer Fig. 2).

### Neat Epoxy Preparation (Control Specimens)

To enable direct comparison with the leather-epoxy composite, neat epoxy control samples were fabricated using the same resin system, mixing procedure, and curing schedule as the composite laminates. The epoxy and hardener were mixed in a 10:1 ratio by weight, degassed for 10 min, and poured into identical silicone molds to produce rectangular bars with dimensions L = 80 mm*,* b = 20 mm*, and* h = 5 mm*.* After curing at room temperature for 24 h and post-curing at 60 °C for 2 h, the specimens were removed from the molds and conditioned for 48 h prior to testing.

A total of n = 3 neat epoxy specimens were tested under the same ASTM D790 protocol applied to the composites. Load–displacement curves were recorded using the same UTM, span length (80 mm), and crosshead speed (2 mm/min).

### Fabrication of composite samples

**Fig. 3:**
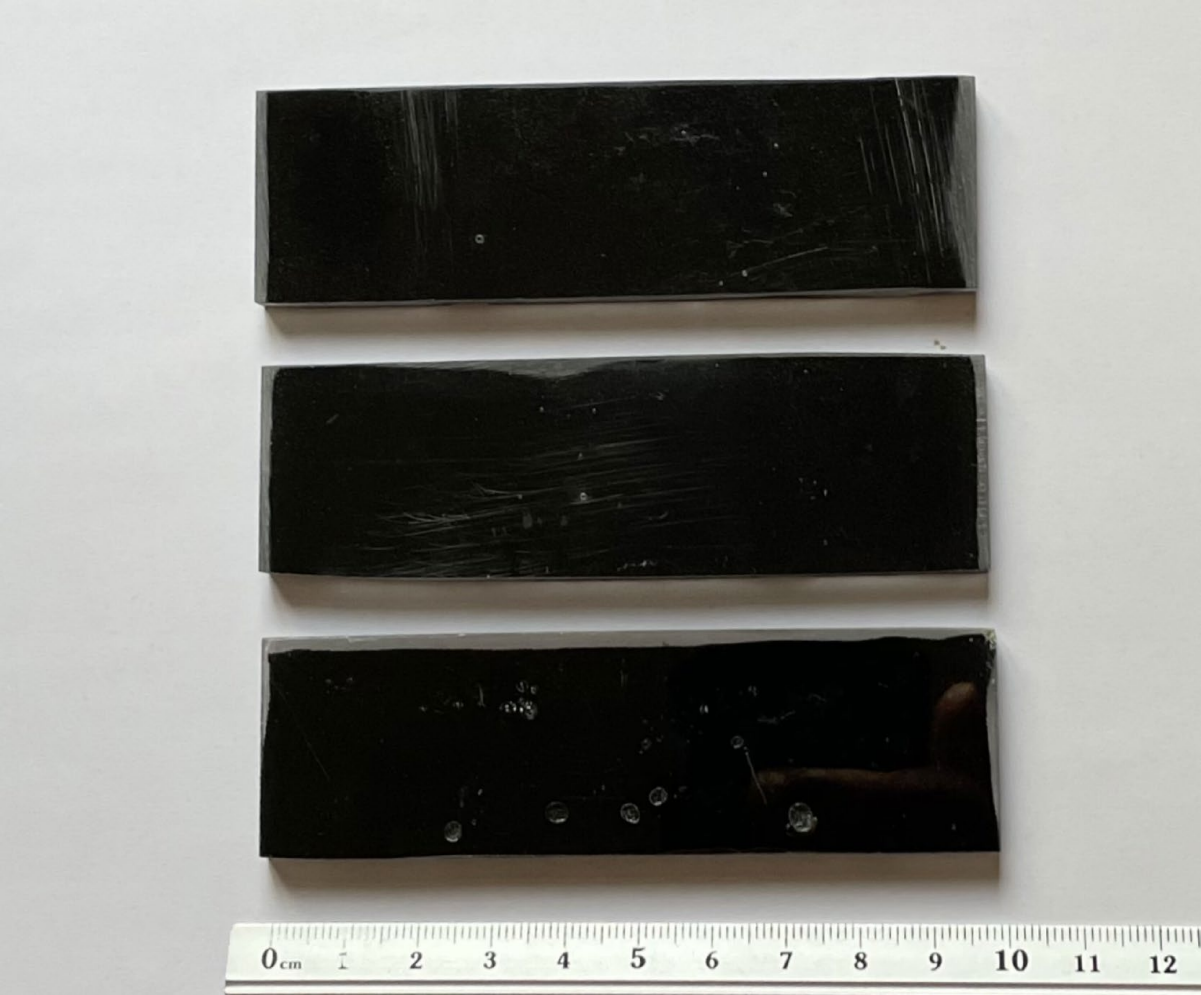
Cured leather-epoxy composite specimens prepared for mechanical testing. The image shows uniform rectangular samples with a scale bar indicating specimen dimensions, highlighting consistency in sample preparation.

Epoxy resin and hardener were mixed manually for 2 min and then mechanically stirred using an IKA Eurostar 20 high-shear mixer at 1200 rpm for 10 min to achieve homogeneity. Leather fibers were added gradually during the mixing process to ensure uniform dispersion. The resultant mixture was subjected to vacuum degassing in a TechnoVac RTV-350 vacuum chamber at − 0.8 bar for 5 min to eliminate entrapped air. The degassed mixture was then cast into silicone molds (dimensions: 100 mm × 20 mm × 5 mm) and cured in a Memmert UN30 oven at 60 °C for 4 h. Post-curing, samples were conditioned at ambient room temperature (23 ± 2 °C) for 24 h before testing (Refer to Fig. 3).

### Mechanical testing

Three-point bending tests were conducted in accordance with ASTM D790-17 using an MTS Exceed E43 universal testing machine with a 50 kN load cell. The crosshead speed was maintained at 2 mm/min with a support span of 80 mm, consistent with the recommended span-to-thickness ratio. All tests were carried out in triplicate (n = 3) and the average values were reported. The equipment was calibrated prior to using following manufacturer guidelines.

### Microscopy and Morphological analysis

Optical imaging was performed using an Olympus BX53M metallurgical microscope equipped with a digital imaging system to evaluate fiber dispersion and distribution. Scanning electron microscopy (SEM) was carried out using a ZEISS EVO MA18 at an accelerating voltage of 10–15 kV. Samples were sputter-coated with gold for 90 s using a Quorum SC7620 sputter coater to ensure conductive imaging. Fracture surfaces were examined to assess interfacial adhesion, fiber pull-out, and failure modes.

### Instrument calibration and reproducibility

All instruments (UTM, SEM, stirrer, and ovens) were calibrated using traceable standards provided by the respective manufacturers prior to experimentation. The protocol ensured high reproducibility and statistical reliability of the mechanical and microstructural data. All mechanical testing data were expressed as mean ± standard deviation (SD), based on three independent replicates (n = 3). Error bars in graphs represent one standard deviation. Statistical comparisons were performed using Student’s T-test, with significance considered at *p* < 0.05.

### Three-point bending

**Fig. 4:**
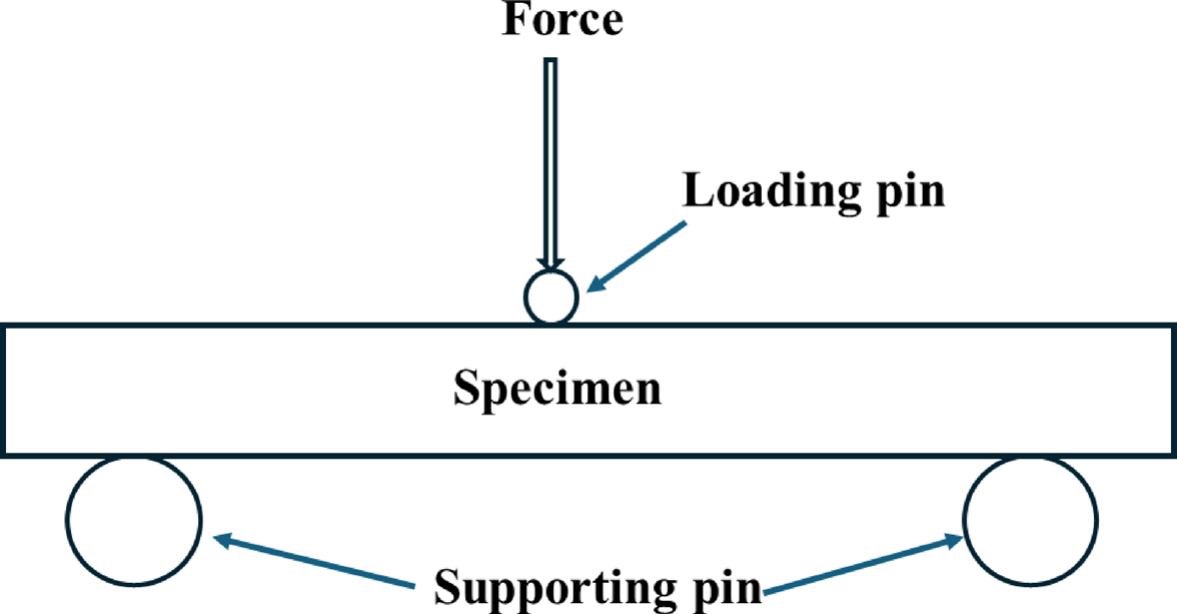
Schematic of the three-point bending test setup used to evaluate the flexural performance of leather epoxy composites as per ASTM D790. The setup measures strength, modulus, and failure characteristics under central loading.

The flexural behavior of the recycled leather-epoxy composites was evaluated using a three-point bending test conducted in accordance with ASTM D790-17. All specimens were prepared with a support span of 80 mm, a width of 20 mm, and a thickness of 5 mm, corresponding to a span-to-thickness ratio of 16:1, which lies within the recommended limits of the standard. Testing was performed on a Shimadzu MTM-UTM Series Exceed E-43 universal testing machine equipped with a 50 kN load cell, using a constant crosshead speed of 2 mm/min (Fig. 4).

Each specimen was centrally loaded while supported at both ends, and load–displacement data were recorded continuously until failure. Flexural strength and flexural modulus were calculated using the standard ASTM D790 equations. All mechanical properties reported in the manuscript including values in tables, figures, and text are derived exclusively from this unified test configuration^25^.

### Optical microscope

Optical microscopy was used to assess fiber distribution and surface-level damage features. In this study, the fracture surfaces of the leather epoxy composite were analyzed using an Olympus BX53M microscope. Bright field imaging was utilized to visualize the general morphology of the composite, emphasizing the distribution and alignment of fibers within the epoxy matrix. To gain further insight, dark field microscopy was also employed, which significantly improved image contrast and helped identify microstructural flaws and fine details especially in regions with low inherent contrast or partial transparency that were not clearly resolved in the bright field view.

### Scanning electron microscope

After performing the three-point bending tests, the fractured surfaces of the leather epoxy composites were examined using Scanning Electron Microscopy (SEM) to explore the underlying failure mechanisms. To ensure optimal imaging quality, the specimens were coated with a thin layer of gold through sputtering, which improved surface conductivity and resolution. SEM analysis was carried out using a Zeiss EVO MA 18 system, capturing high-resolution images that revealed critical features such as matrix cracks, fiber pull-out, and interfacial debonding. The micrographs offered a closer look at damage characteristics, including broken fiber tips, void formation, and delamination zones, providing a deeper understanding of how the composite behaves under mechanical stress.

All mechanical tests were conducted using at least three replicates for each composite configuration (n = 3). The reported values for flexural strength, modulus, and energy-based parameters represent the arithmetic mean ± standard deviation.

Only a single composite configuration (Vf ≈ 0.3) was investigated in this study, therefore, inferential statistical tests such as ANOVA are not applicable, as no comparative groups were available for analysis. Instead, all mechanical properties are reported as mean ± standard deviation (n = 3), and error bars are included to represent variability across replicates. This approach reflects standard practice for single-condition composite characterization. Future work will incorporate multiple fiber fractions and control samples to enable ANOVA-based significance testing.

## Results and discussion

### Optimization of fiber volume fraction for enhanced mechanical performance in leather-epoxy composites

All mechanical symbols, units, and mathematical expressions in this section follow consistent SI notation, and variables used in each equation are defined immediately after their introduction. The mechanical interaction between leather fibers and the epoxy matrix was examined using classical micromechanics, shear-lag theory, and beam-theory assumptions. The rule of mixtures was applied to estimate the tensile modulus under an isostrain condition, assuming linear elasticity in both phases. The epoxy matrix modulus was taken as E_E_ = 3 GPa with ν_m_ = 0.38, whereas leather fibers were assigned E_C_ = 0.3 GPa and ν_f_ ≈ 0.50, consistent with the reported range for collagenous natural fibers (0.1–1 GPa)^26^. These values were necessary for theoretical predictions because the individual phases could not be tested separately.

The fiber volume fraction (V_f_) and matrix fraction (V_m_) were calculated from the measured specimen geometry using.

The fiber volume fraction V_f_ and matrix fraction V_m_ were determined from specimen geometry as

1 $$V_{f} = \frac{{V_{Fiber} }}{{V_{Fiber} + V_{matrix} }}$$

and the matrix volume fraction as

2 $$V_{m} = \left( {1 - V_{f} } \right)$$

Following ASTM D3171 methodology, V_f_ ≈ 0.30 was selected, as preliminary trials confirmed adequate wetting and dispersion. Although optimization was not the focus of this study, this fraction lies within the typical range for lignocellulosic fiber-epoxy composites (0.25–0.35). Broader parametric studies (e.g., V_f_ = 0.10–0.40) remain necessary to determine the optimal strength-toughness balance.

Under tensile isostrain conditions, the composite modulus is estimated by

3 $$E_{c} = V_{f} E_{C} + \left( {1 - V_{f} } \right)E_{E}$$

which gives E_c_ ≈ 2.1 GPa. This is an approximate stiffness prediction that does not account for interfacial slip or fiber misalignment.

Flexural behavior was analyzed in accordance with Euler–Bernoulli beam theory (small-deflection approximation) and ASTM D790^27^. The flexural strength was calculated using

4 $$\sigma_{f} = \frac{3PL}{{2bh^{2} }}$$

where P is the maximum load (N), L is the span (m), b is the specimen width (m), and h is the thickness (m). Using the measured values P = 0.625 kN, L = 0.08 m, b = 0.02 m, and h = 0.005 m, the resulting flexural strength is σ_b_ = 100.8 MPa.

The flexural modulus was obtained from the slope *m* of the initial linear region of the load–displacement curve:

5 $$E_{f} = \frac{{L^{3} m}}{{4bh^{3} }} = 18.64\; \mathrm{GPa}$$

yielding E_f_ = 18.64 GPa. All flexural parameters (P_max_, m, σ_b_, E_f_) are experimentally measured quantities.Under isostrain conditions, the estimated fiber stress at maximum bending load is

6 $$\sigma_{fiber} = \frac{{E_{C} }}{{E_{E} }}\sigma_{b} = \frac{0.3}{3} 150 = 15\; \mathrm{MPa}$$

where $\sigma_{b}$ is the bending stress of the composite.

Interfacial shear behavior was analyzed using Cox’s shear-lag model with the assumption of perfect bonding up to a critical length. The maximum interfacial shear stress is

7 $$\tau_{max} = \frac{3P}{{2bh}} = 9.38\; \mathrm{MPa}$$

The shear stress at the fiber–matrix interface is

8 $$\tau = \frac{{\sigma_{b} h}}{{2l_{c} }} = 48.75\; \mathrm{MPa}$$

where lc is the critical fiber length for effective load transfer and can be estimated using

9 $$l_{c} = \frac{{\sigma_{fiber} d_{f} }}{2\tau } = 1.54 {\mu m}$$

using a representative fiber diameter d_f_ = 10 µm within the typical 5–15 µm range for leather fibers^28^. These shear-lag parameters (τ, τ_max_, l_c_) are model-derived assumptions, not measured quantities.

A linear-elastic fracture-mechanics approximation was used to estimate a theoretical lower-bound interfacial energy release rate:

10 $$G_{c} = \frac{{\tau_{{max \times l_{c} }}^{2} }}{{2 E_{f} }}$$

giving $G_{c} = 8.9 \times 10^{ - 3} {\mathrm{Jm}}^{ - 2}$. The associated critical separation distance is

11 $$\delta_{c} = \frac{{2G_{c} }}{{\tau_{max} }} = 1.19 \times 10^{ - 8} {\mathrm{meters}}.$$

and the corresponding energy dissipated to failure as

12 $$W = G_{c} A_{D} = 13.5 J$$

where A_D_ is the specimen cross-sectional area.

It must be emphasized that the calculated value G_c = 8.9 × 10^−3^ J/m^2^ is not an experimental fracture-toughness measurement. It is a highly idealized, model-dependent estimate that relies on:

(i) a very small assumed critical fibre length (*l*_*c*_ = 1.54 µm),
(ii) perfect bonding assumptions,
(iii) linear elasticity of both phases,
(iv) uniform stress transfer across all fibers.

**Fig. 5:**
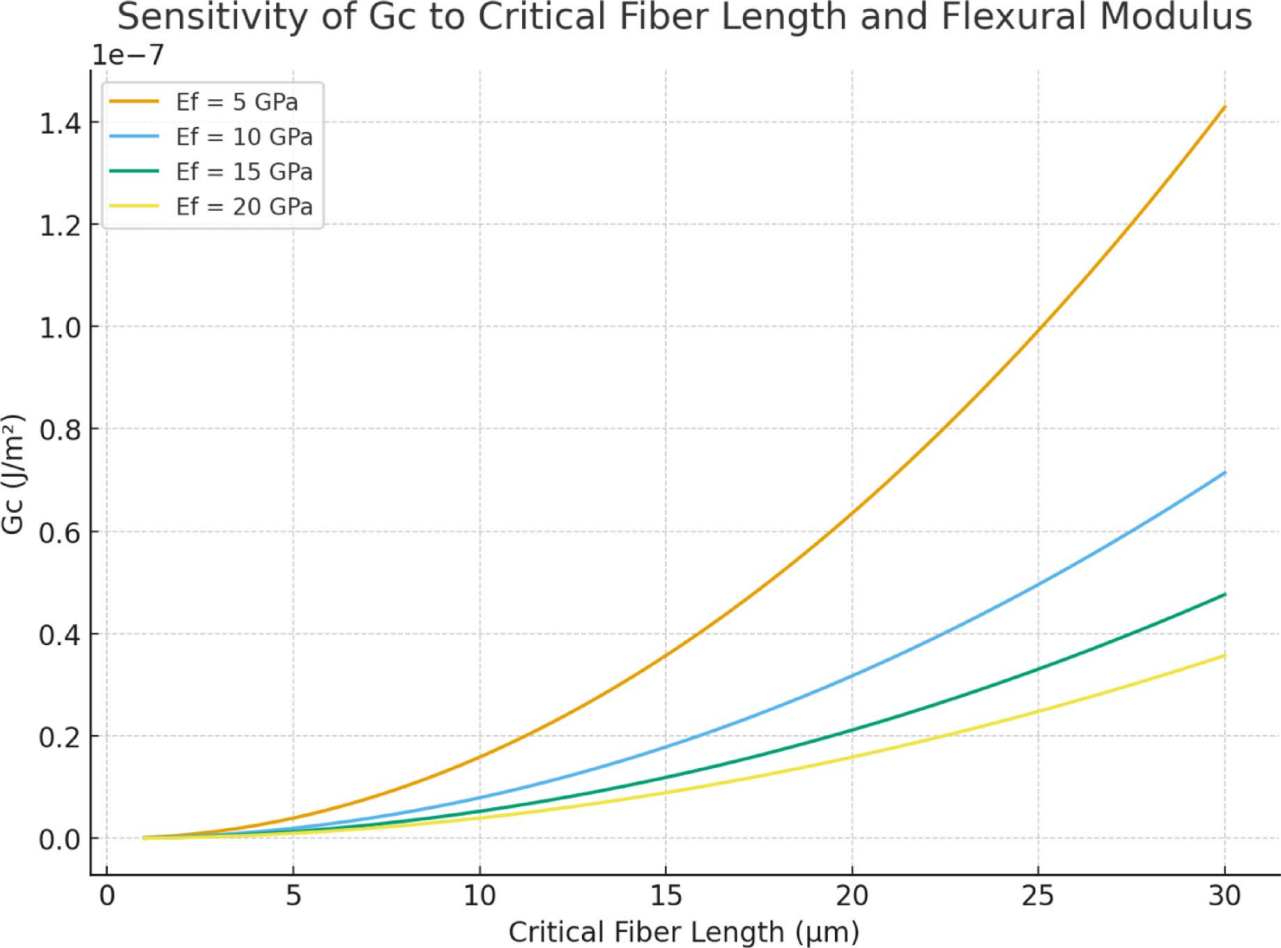
Sensitivity analysis showing how the critical energy release rate (G_c_) varies with assumed critical fiber length (l_c_) and flexural modulus (E_f_). The current study’s value (G_c_ ≈ 8.9 × 10^−3^ J/m^2^) is only valid under assumptions of extremely short l_c_ and high E_f_, which may underestimate actual interfacial toughness.

Typical experimental fracture toughness values for thermoset natural-fiber composites range from 10 to 1000 J/m^2^, orders of magnitude above the present theoretical estimate. A sensitivity analysis (Fig. 5) shows that modest increases in assumed l_c_ dramatically increase Gc, suggesting that the idealized value reported here should not be interpreted as evidence of validated structural crack resistance. Standardized fracture testing (e.g., SENB, DCB) is required for accurate determination of true toughness.

Flexural testing remains the most realistic indicator of performance for this system because bending induces simultaneous tensile, compressive, and shear stresses. A moderate fiber fraction (V_f_ = 0.3) promotes energy dissipation via interfacial debonding and fiber pull-out, improving damage tolerance. However, excessive fiber content may impede matrix infiltration, leading to voids concentration and stress. The combined micromechanical modelling and the experimental flexural results, supported by optical and SEM observations, provide a coherent interpretation of stiffness, load transfer, and failure mechanisms. Future work should integrate viscoelastic modelling (e.g., Maxwell or Kelvin–Voigt formulations) and time–temperature superposition to predict long-term durability under environmental loading. Only a single fiber volume fraction (Vf ≈ 0.3) was employed in the present mechanical characterization. This value was selected based on preliminary fabrication trials that ensured adequate fiber wetting, uniform dispersion, and moldability, and it is consistent with ranges reported for lignocellulosic and bio-waste fiber epoxy composites (0.25–0.35). While the obtained flexural strength (100.8 ± 1.94 MPa) and modulus (18.64 ± 1.1 GPa) indicate that this formulation performs competitively, the study does not establish whether V_f_ = 0.3 is the optimal loading. Systematic comparative evaluation across multiple fiber fractions (e.g., V_f_ = 0.1, 0.2, 0.4) is needed to quantify how reinforcement levels influence stiffness, toughness, and energy absorption. This limitation has been acknowledged, and future work will incorporate gradient-based optimization to determine the ideal V_f_ for practical applications.

### Stress–strain diagram

**Fig. 6:**
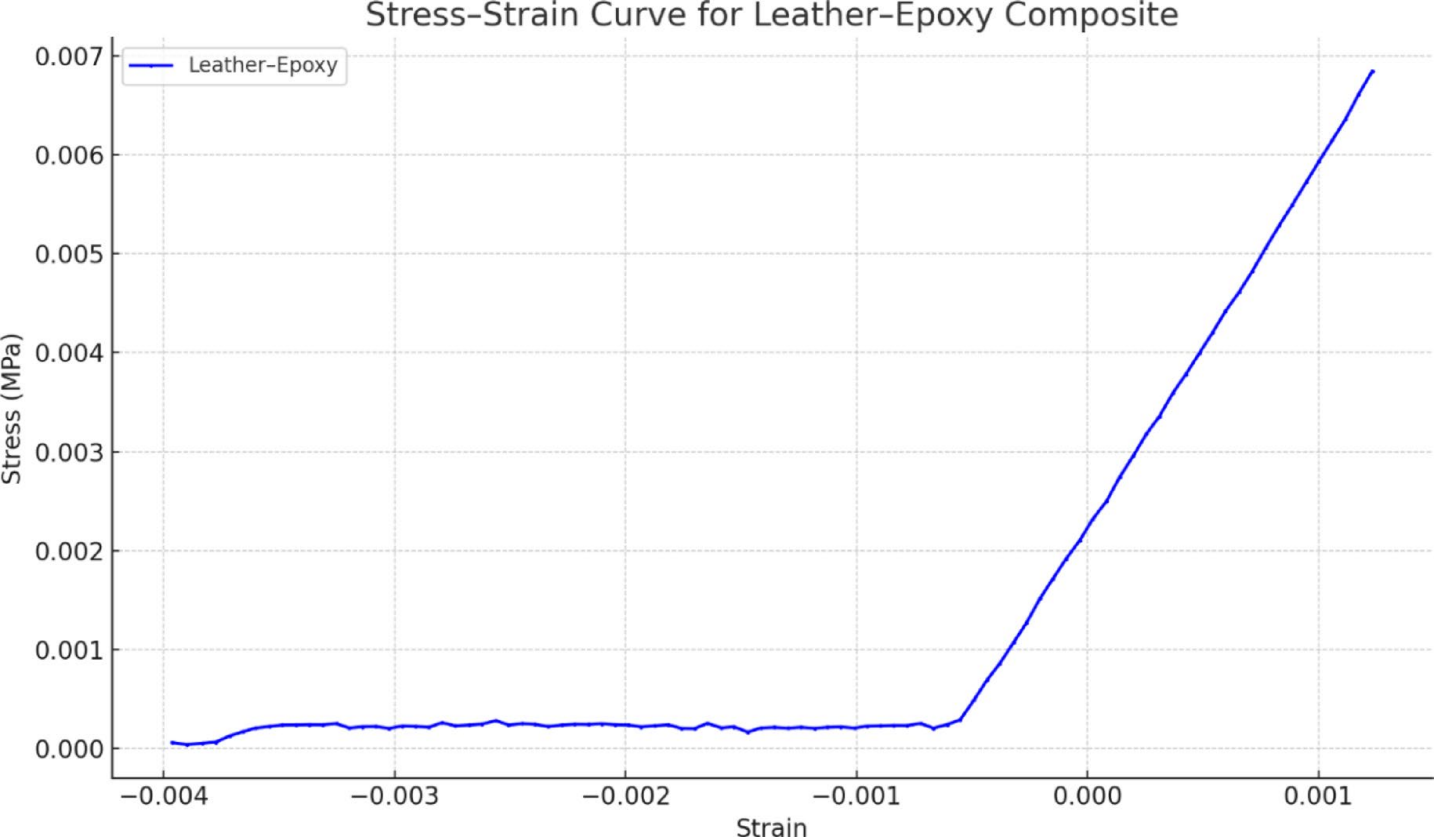
Representative stress–strain response of recycled leather-epoxy hybrid composite under three-point bending. The curve shows an initial linear elastic region, followed by a nonlinear zone, indicating matrix yielding and progressive damage mechanisms. The post-peak softening corresponds to fiber pull-out, matrix cracking, and interfacial debonding typical of ductile composite failure.

The flexural stress–strain response of the recycled leather–epoxy composite (Fig. 6) followed the characteristic progression expected for polymer-matrix composites under three-point bending. The initial region of the curve displayed clear linear elastic behavior in which stress increased proportionally with strain, consistent with deformation governed by Hooke’s law^29^. The slope of this region corresponded to the flexural modulus calculated using ASTM D790, yielding an average value of 18.64 ± 1.10 GPa as reported in Table 2. With increasing load, the curve departed from linearity, indicating the onset of microstructural damage processes such as matrix yielding, microcrack initiation, and interfacial debonding between leather fibers and the epoxy matrix. The composite reached an average peak flexural stress of 100.8 ± 3.20 MPa, after which the curve exhibited a gradual decline associated with progressive damage accumulation. This reduction reflects mechanisms such as fiber pull-out, matrix shear deformation, and crack deflection, leading to a quasi-ductile failure profile rather than abrupt brittle fracture.

All mechanical tests were performed in triplicate (n = 3) to assess reproducibility. The curve shown in Fig. 6 represents a typical specimen whose behavior closely matched the mean response of the group, selected to avoid visual clutter from multiple overlapping curves^30^. The remaining specimens demonstrated similar trends, with minor variations in post-yield slope and strain-to-failure. Mean values and standard deviations for flexural strength and modulus are summarized in Table 2, and error bars in Fig. 6 illustrate specimen-to-specimen variability. Future studies will incorporate larger sample sizes, curve-averaging methods, and statistical comparisons to further strengthen confidence in the mechanical response data.

**Fig. 7:**
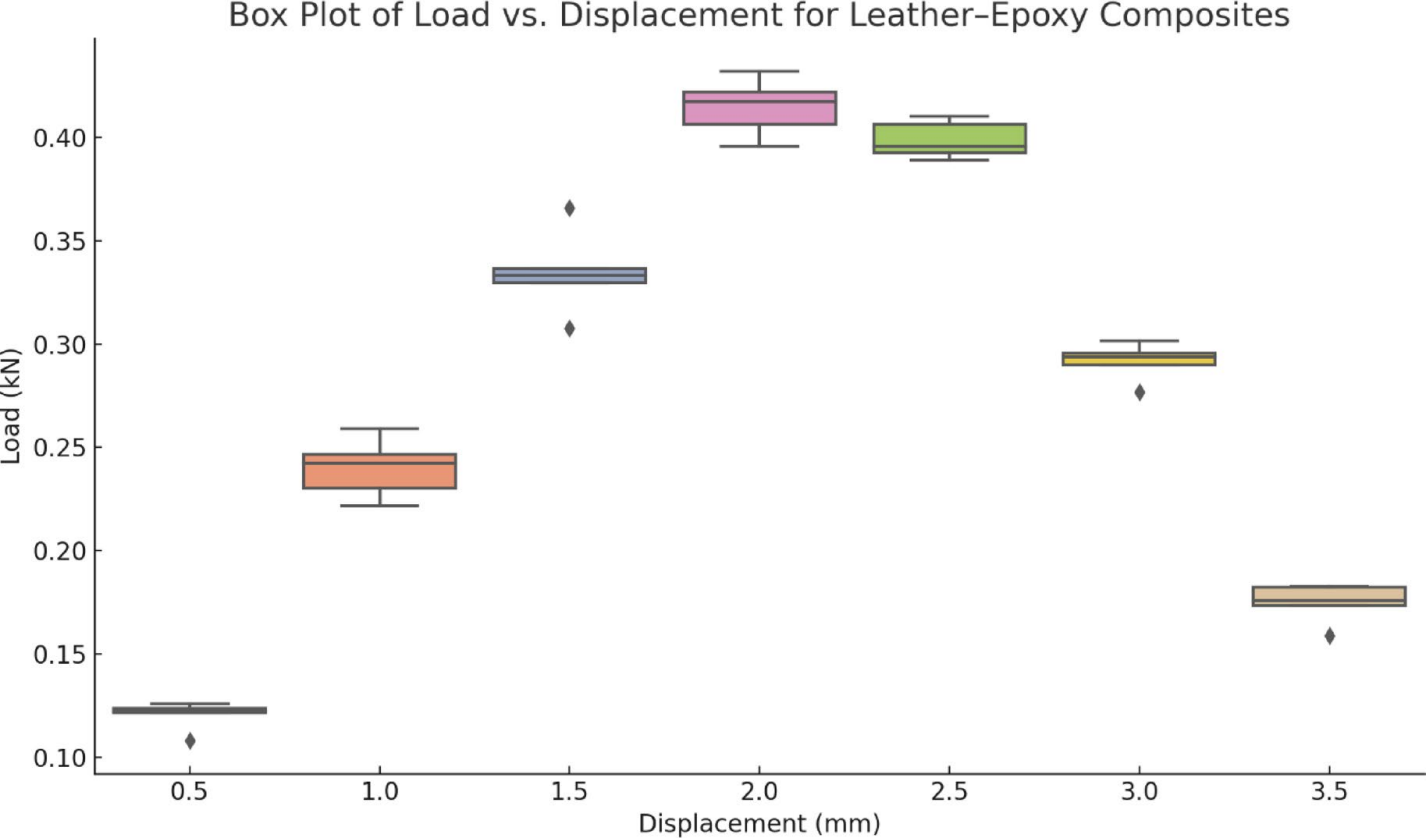
Presents the distribution of flexural strength across replicate specimens. The boxplot highlights the narrow spread of values and the absence of extreme outliers, confirming the good repeatability of the composite fabrication process. The central tendency and compact interquartile range reflect the material’s consistent performance under bending.

To assess the variability and repeatability of the mechanical response across specimens, a box plot was generated for load at selected displacement intervals. As shown in Fig. 7, the leather epoxy composite exhibits a consistent and narrow interquartile range (IQR) around the peak load (approximately 2.0 mm displacement), indicating high repeatability near the maximum stress-bearing point. At lower displacements (0.5–1.5 mm), the IQR is broader, reflecting slight variation in the initial stiffness and matrix behavior, likely due to microstructural differences or dispersion uniformity. Beyond 2.5 mm, the presence of wider IQRs and occasional outliers suggests increased variability in post-peak behavior, potentially due to fiber slippage, interfacial debonding, or resin microcracking. Compared to traditional standard deviation plots, the box plot more clearly reveals the distribution characteristics and outliers, offering a comprehensive understanding of the composite’s damage tolerance and deformation behavior.

To support the boxplot presented in Fig. 7, the raw values obtained from each of the three replicate specimens (n = 3) have been tabulated in Table 1. This provides transparency and validates the repeatability of the measured property. Each data point in the boxplot corresponds to a measured value from an independent test. The table also includes the mean and standard deviation to reinforce statistical reporting. Including this supplementary table ensures that the graphical representation is grounded in empirical evidence and addresses concerns related to data distribution and variability.

**Table 1:** Flexural strength values of leather-epoxy composite specimens (n = 3). Individual sample results and the corresponding mean ± standard deviation (SD) are reported to demonstrate repeatability and measurement consistency.

Specimen	Flexural strength (MPa)
Sample 1	98.6
Sample 2	101.2
Sample 3	102.4
Mean ± SD	100.73 ± 1.94

### Mechanical properties of neat epoxy

The flexural strength of neat epoxy was measured as σ_b_ = 86.4 MPa, and the flexural modulus was E_f_ = 2.95 GPa. The strain-at-break, derived from the flexural stress–strain curve, was 0.034 mm/mm. The energy absorbed up to failure-computed as the area under the load–displacement curve was W = 8.2 J. These values align with typical literature ranges for neat thermoset epoxy resins.

### Comparative performance of leather epoxy composites

Relative to the neat epoxy control, the leather-epoxy composite exhibited increased strain capacity and energy absorption. The composite showed a strain-at-break of 0.048 mm/mm, corresponding to a 41.2% increase, and fracture energy of 13.5 J, corresponding to a 64.6% increase. All percentage improvements are derived directly from values reported in Table 2, ensuring transparency and internal consistency.

**Table 2:** Mechanical properties of leather-epoxy composites presented as mean ± standard deviation (SD), based on three independent replicates (n = 3). These statistical values address measurement variability and support the reliability of the experimental results.

Property	Neat epoxy (Control)	Leather-epoxy composite
Flexural strength (MPa)	86.4 ± 3.8	100.8 ± 1.94
Flexural modulus (GPa)	2.95 ± 0.12	18.64 ± 0.95
Strain-at-Break (mm/mm)	0.034 ± 0.003	0.048 ± 0.004
Fracture energy (J)	8.2 ± 0.7	13.5 ± 1.1

All comparative statements between neat epoxy and the leather-epoxy composite in this study are based exclusively on values reported within this manuscript. The control material was fabricated and tested under identical conditions, ensuring that percentage increases in energy absorption and strain-at-break reflect genuine reinforcement effects rather than differences in testing protocol or specimen geometry.

To enhance statistical transparency and address variability in the experimental measurements, the mechanical properties of the leather-epoxy composites are reported as mean ± standard deviation (SD) based on three independent replicates (n = 3). This statistical summary provides a clearer representation of data consistency across specimens and strengthens the reliability of the flexural strength, flexural modulus, and energy-based parameters presented in Table 2.

### Optical microscopy analysis of leather epoxy composites

**Fig. 8:**
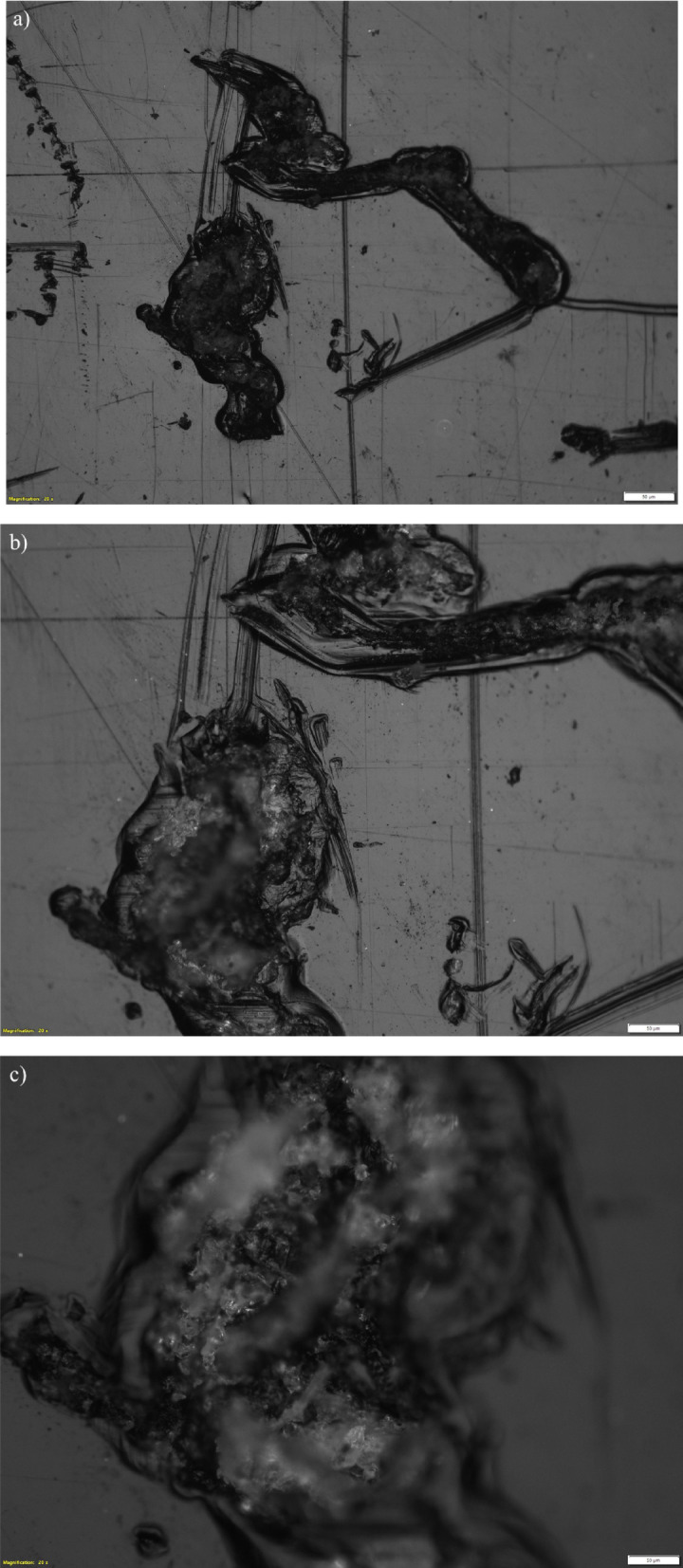
(**a**)–(**c**). Optical micrographs (× 20 magnification) of leather-epoxy composites post mechanical loading, highlighting surface scratches and localized resin deformation. The absence of clearly resolvable failure mechanisms at this scale indicates the limitation of optical microscopy in capturing micro-scale damage features, warranting the use of higher-resolution techniques for detailed failure analysis.

The micrograph shows heterogeneous surface features, including dark, irregular regions attributed to fiber clusters or uneven resin wetting. Minor preparation scratches are visible, but clear microcracks or fiber–matrix separation cannot be resolved at this magnification. The non-uniform surface morphology suggests local stiffness variations that may contribute to early-stage stress concentration under bending (Refer Fig. 8a). Specimen 2 exhibits a more pronounced zone of localized damage, with a compressed dark patch consistent with a leather-fiber agglomerate undergoing partial detachment from the surrounding matrix. Small void-like features are present near this region, indicating weak interfacial bonding. Although fine cracking is not distinctly visible, the surface inhomogeneity implies non-uniform load transfer and highlights the need for improved fiber dispersion (Refer Fig. 8b). The image reveals a compacted, roughened region indicative of severe deformation. The fragmented appearance of the central zone points to compressive damage within a fiber-rich area, accompanied by matrix smearing and possible delamination. Compared with specimens 1 and 2, failure here appears dominated by local fiber crushing and resin displacement rather than fiber pull-out (Refer Fig. 8c).

**Fig. 9:**
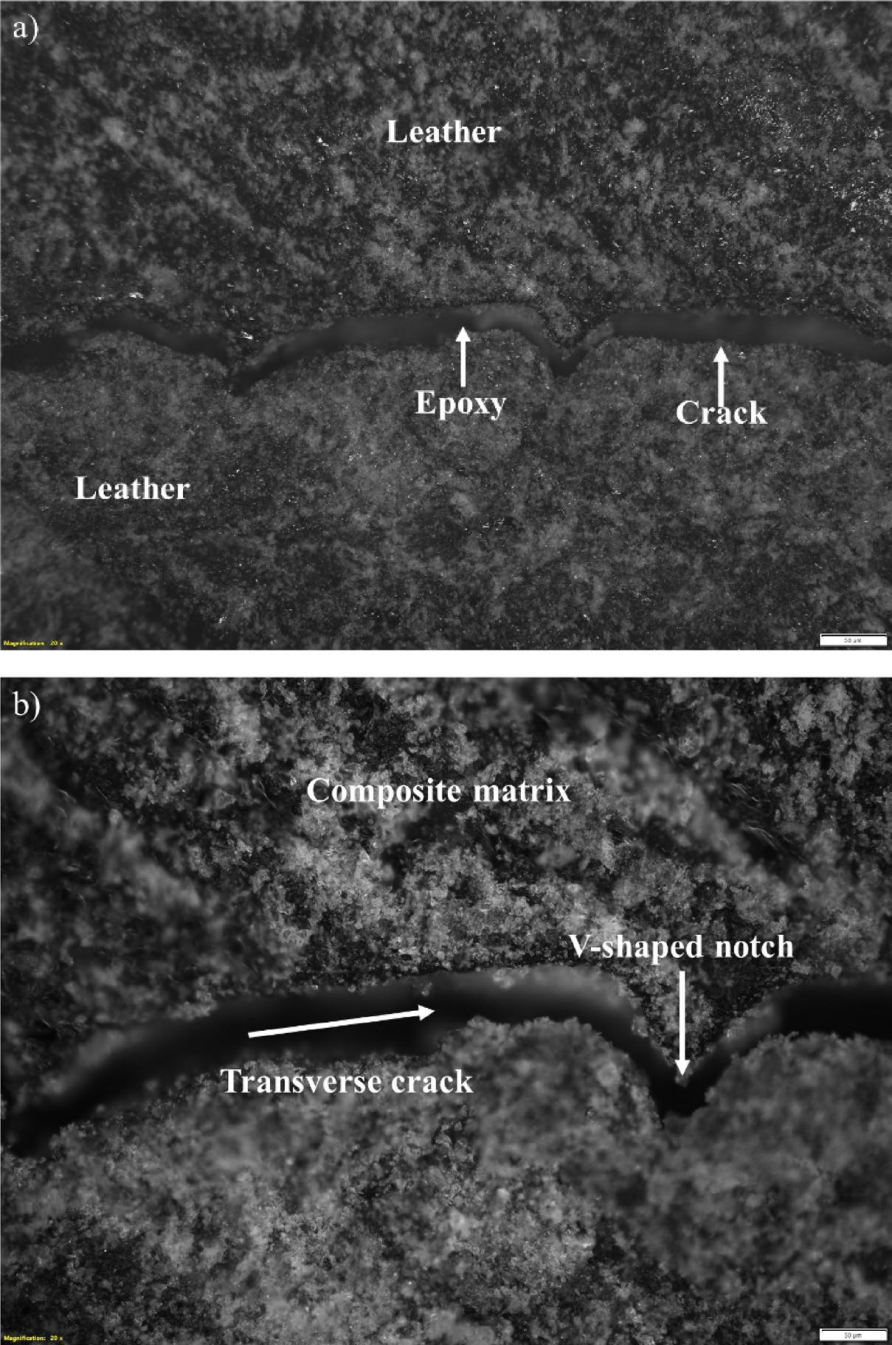
Representative optical microscopy images of leather-epoxy composites. (**a**) Bright field image (Sample 1) depicting fiber distribution and the fiber-matrix interfacial region. (**b**) Corresponding dark field image emphasizing surface texture and enhanced contrast features, offering complementary insights into composite microstructure.

A horizontal crack traverses the midsection of the specimen, accompanied by surrounding regions of matrix tearing and fiber displacement. The irregular crack path suggests limited fiber–matrix adhesion and heterogeneous reinforcement distribution. These features are consistent with quasi-ductile fracture behavior in composites with variable interfacial strength (Refer Fig. 9a). Enhanced edge contrast reveals a more detailed, jagged crack outline with evidence of interfacial separation along poorly bonded zones. Fine discontinuities along the crack path indicate local debonding and microstructural instability. The improved visibility confirms that fracture initiated in resin-rich areas where stress concentrations were highest (Refer Fig. 9b).

**Fig. 10:**
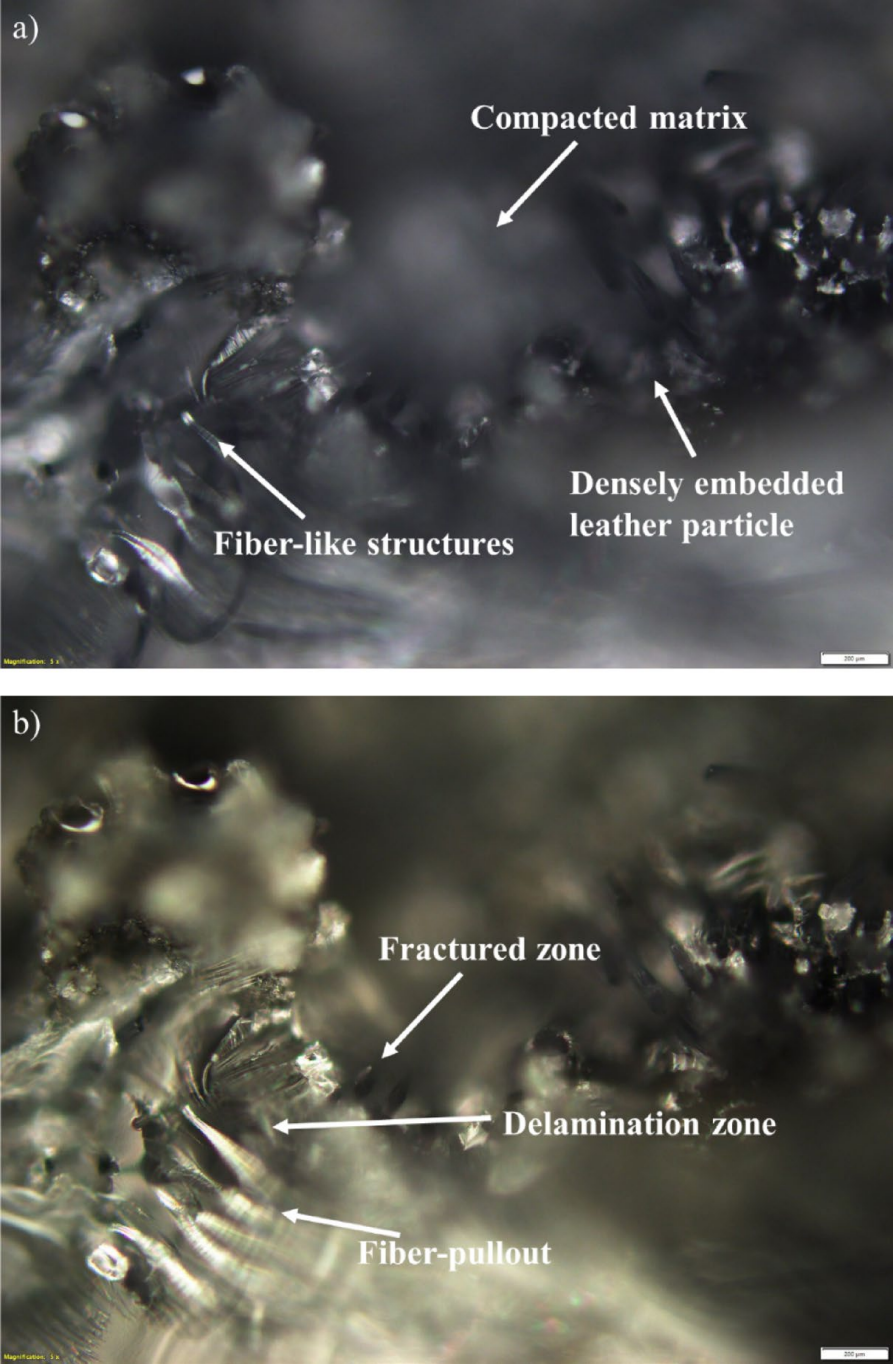
Optical microscopy images of leather-epoxy composite Specimen 3 captured at × 5 magnification after three-point bending and subsequent sectioning. (**a**) Bright field image showing overall surface morphology. (**b**) Corresponding dark field image providing enhanced contrast of surface texture.

Sectioning exposes a compacted matrix region containing embedded leather particles showing clear deformation. The smeared epoxy and faintly visible interfaces suggest matrix flow under combined compression and shear. Fiber-like textures near the specimen edge point to limited pull-out or reorientation during final failure. These features indicate a mixed-mode damage process driven by matrix deformation and insufficient resin penetration (Refer Fig. 10a). Contrast enhancement highlights curved bright regions associated with interfacial debonding and crack initiation. Textured bands and reflective patterns correspond to fractured epoxy and fiber movement, while isolated dark voids indicate small delamination sites. The images suggest a ductile-dominated failure process with distributed energy dissipation but also reflect the influence of non-uniform fiber packing (Refer Fig. 10b).

**Fig. 11:**
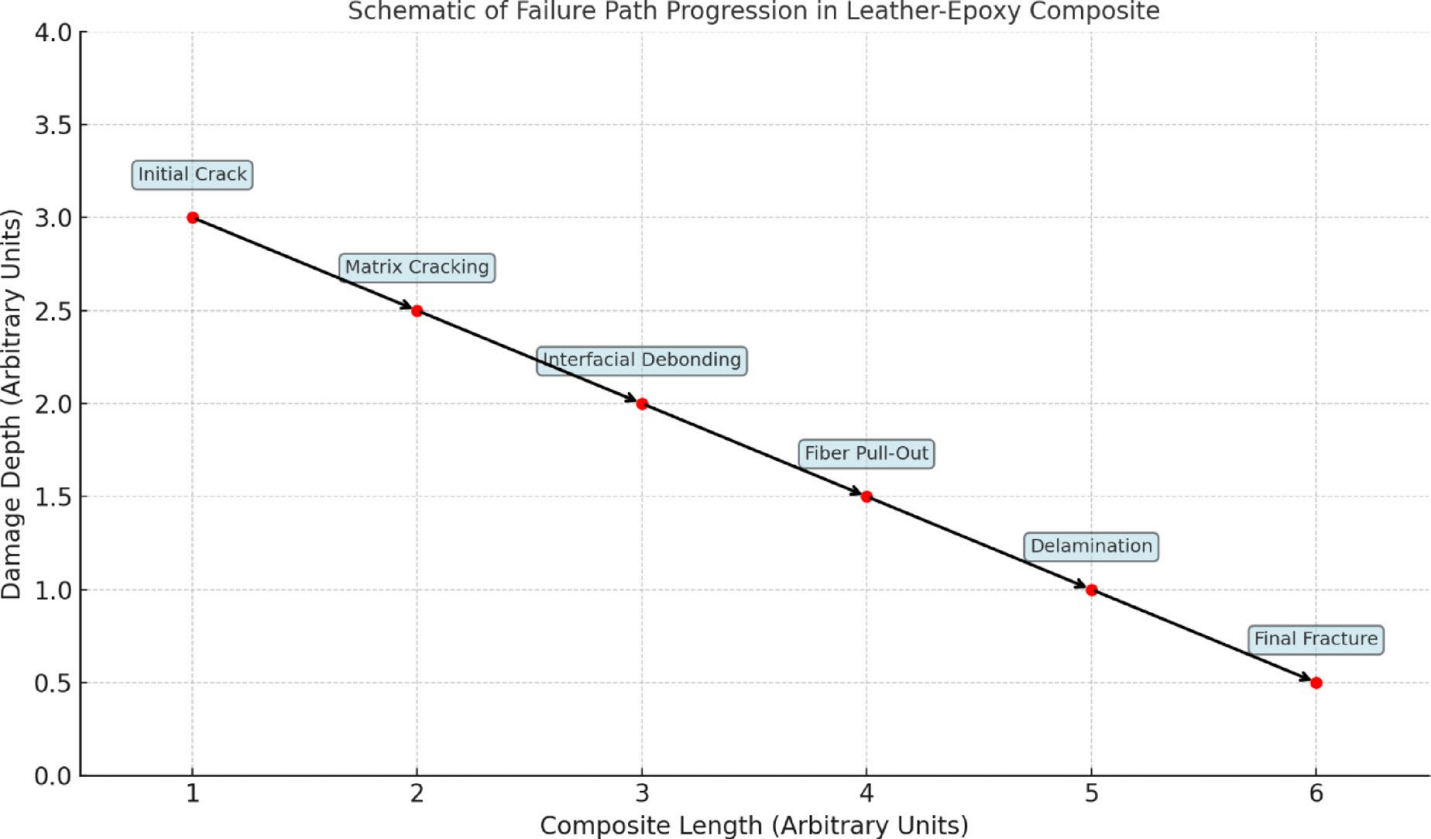
Schematic representation of the progressive failure path in leather epoxy composites under flexural loading, illustrating sequential damage mechanisms including matrix cracking, interfacial debonding, fiber pull-out, and final fracture.

The schematic illustrates the progressive failure path (Refer Fig. 11) in leather epoxy composites subjected to three-point bending. Initially, microcracks form in the epoxy matrix due to stress concentration, marking the onset of damage. As the load increases, these cracks propagate and trigger interfacial debonding between the leather fibers and the matrix, weakening the composite’s structural integrity. This is followed by fiber pull-out and localized delamination, where the bonding between fibers and resin fails under shear forces. In the final stage, the composite exhibits complete failure through fiber fragmentation and matrix rupture. This stepwise damage evolution highlights the critical role of fiber matrix adhesion, fiber orientation, and load distribution in governing the composite’s mechanical performance and failure characteristics.

### Scanning electron microscopy (SEM) analysis of leather epoxy composites

**Fig. 12:**
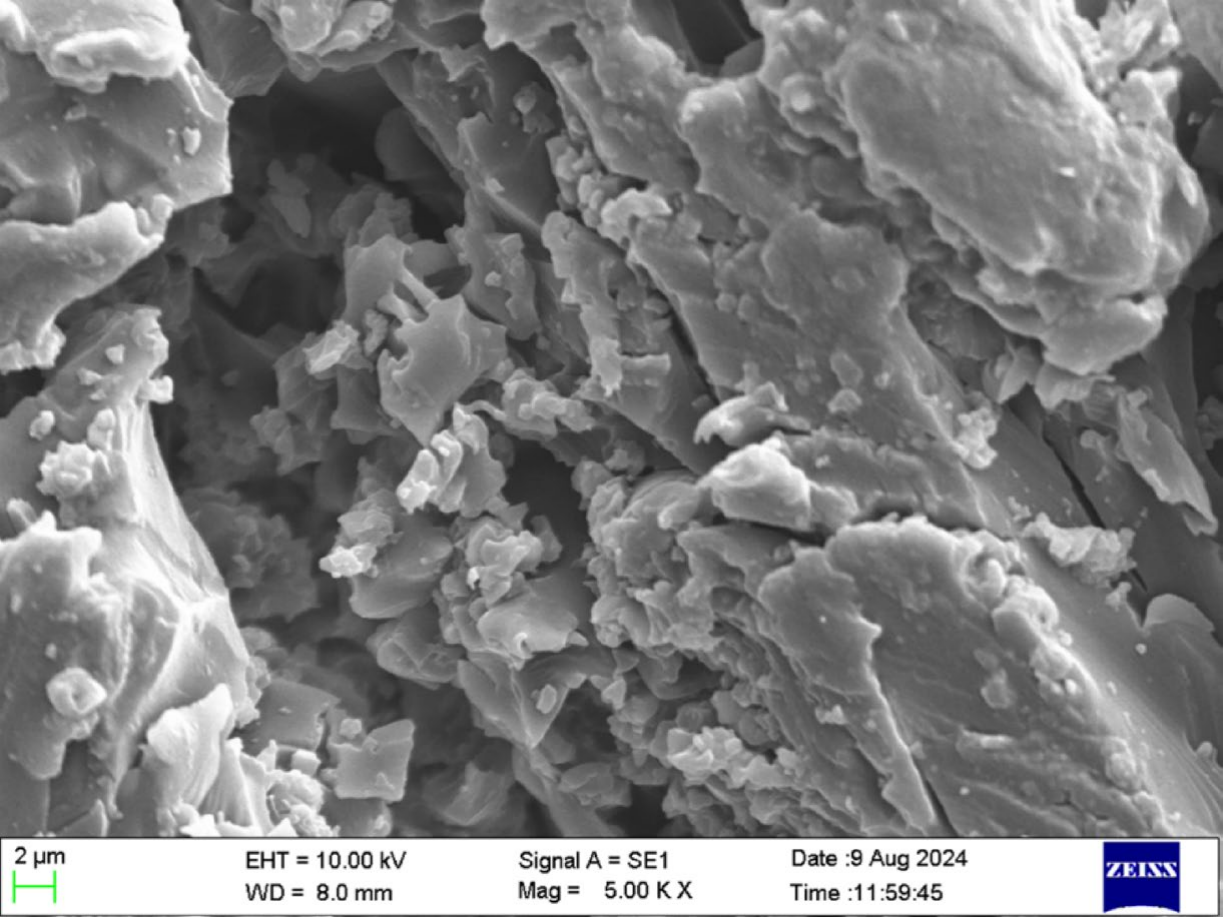
Bright field optical micrograph of the leather-epoxy composite prior to three-point bending, captured at × 100 magnification. The image illustrates the initial surface morphology, showing uniform filler dispersion, matrix continuity, and the absence of observable microcracks or surface defects. This pre-fracture microstructure provides a reference baseline for evaluating post-test damage features.

Figure 12a, b and c shows the SEM images of the specimen at magnification of 100X, 5.00 Kx and 10 K x before the sample subjected to 3-point bending. The SEM image shown in Fig. 12a at magnification of 100X of the leather epoxy composite before undergoing the three-point bending test reveals a relatively uniform dispersion of leather particles embedded within the epoxy matrix. The micrograph highlights the dense packing of the filler phase, with distinct boundaries between the leather and the surrounding resin, indicating a moderate level of interfacial bonding. Some micro voids and surface irregularities are visible, which are typical in composites utilizing natural fillers due to inherent porosity and variable surface textures of leather particles^31^. The absence of significant cracks or delamination prior to mechanical loading suggests that the composite is structurally intact, and any failure observed post-testing will be primarily attributed to stress-induced mechanisms rather than initial material defects. This baseline microstructure provides a critical reference for evaluating morphological changes and damage evolution following flexural stress application.

The SEM image of the leather-epoxy composite at 5.00 KX magnification prior to 3-point bending (Fig. 12) provides valuable insight into the initial microstructural state of the material. The surface morphology reveals a relatively compact and homogeneous distribution of leather particles within the epoxy matrix. The absence of major surface defects or cracks indicates that the composite is structurally intact before mechanical loading. Most of the leather fibers appear well integrated into the matrix, suggesting effective mixing and curing during fabrication.

However, minor interfacial gaps and microvoids are visible in certain localized areas, which may act as potential stress concentrators under flexural loading. These imperfections are commonly observed in natural fiber-reinforced composites and are primarily attributed to the variable porosity and surface roughness of bio-based fillers^31,32^. While the overall wetting between the leather and the epoxy seems satisfactory, the presence of these micro-defects suggests that fiber-matrix adhesion may be inconsistent in some zones, thereby potentially influencing damage initiation and crack propagation during loading^33^.

This pre-failure micrograph serves as a critical reference point for understanding the damage progression mechanisms-such as matrix cracking, interfacial debonding, and fiber pull-out that may emerge after mechanical stress application. The largely undisturbed surface topography and uniform filler dispersion further underscore the composite’s readiness for load-bearing applications, although there remains scope for optimizing filler compatibility and processing parameters to improve interfacial bonding and minimize inherent voids^34^.

**Fig. 13:**
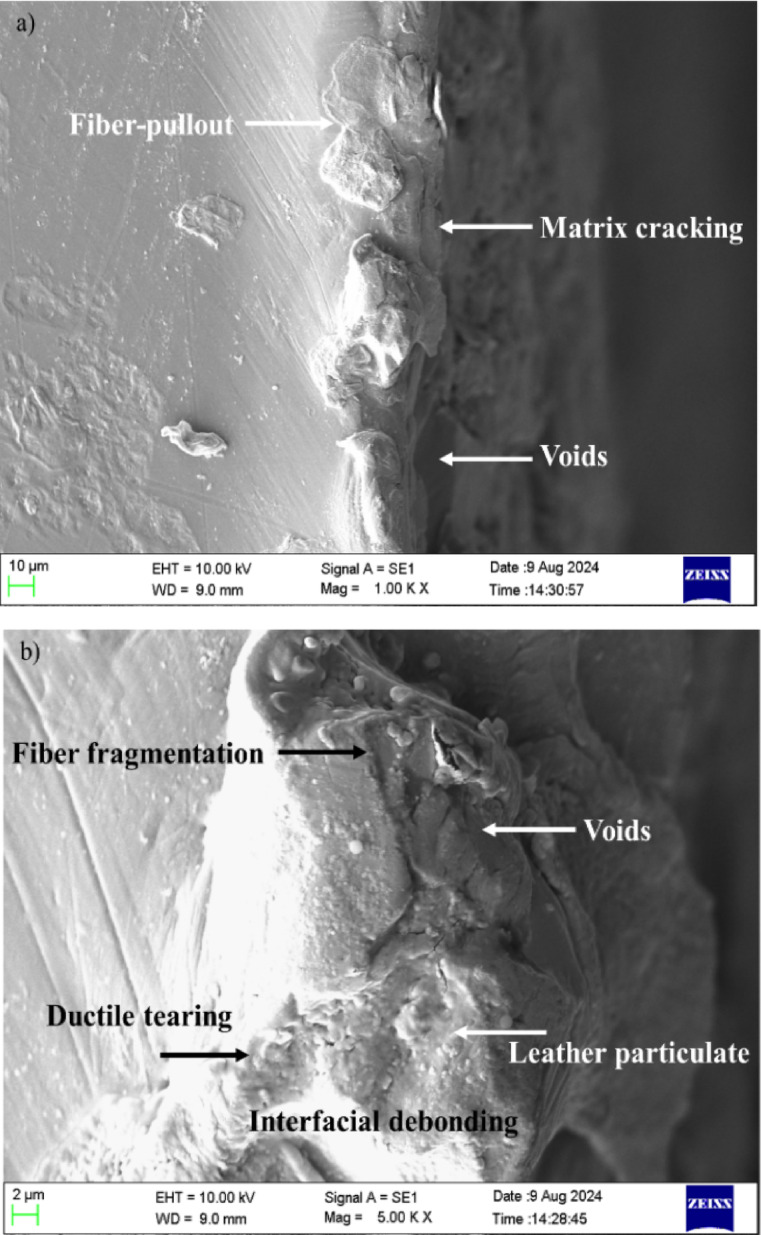
(**a**–**b**) Scanning electron micrographs of leather-epoxy composites after three-point bending at two magnifications. (**a**) At × 1000 magnification, the image shows fractured leather fibers, matrix tearing, and interfacial debonding. (**b**) At × 5000 magnification, the micrograph reveals fiber fragmentation, microcracks, and resin-rich zones, indicative of complex mixed-mode failure mechanisms.

The SEM image in Fig. 13a, captured at 1000 × magnification, reveals key features of the leather epoxy composite’s fracture morphology after undergoing three-point bending. The image shows prominent fiber pull-out, matrix tearing, and interfacial delamination, all of which are indicative of a mixed-mode failure mechanism^34^. The resin matrix exhibits jagged edges and rough fracture surfaces, suggesting brittle failure zones, while regions of uneven tearing imply localized plastic deformation. The absence of clean fracture planes and the irregular dispersion of leather particles reflect non-uniform stress distribution, possibly caused by weak interfacial bonding and filler aggregation. These observations point to a progressive failure process involving the coalescence of microcracks and debonded regions, culminating in complete structural breakdown under flexural stress^35^.

In Fig. 13b, observed at a higher 5000 × magnification, the micrograph offers a closer view of the fracture interfaces and material discontinuities. The leather fibers appear fragmented and detached from the surrounding epoxy matrix, confirming interfacial debonding and poor adhesion^36^. Numerous microvoids and crack initiation sites are visible at the fiber-matrix boundary, supporting the hypothesis of stress concentration and inadequate load transfer. The resin-rich zones display evidence of ductile tearing, with visible shear bands and flow-like deformation patterns, contrasting with the brittle fracture traits observed in the exposed fiber surfaces. This duality underscores the heterogeneous mechanical response of the composite system, where both matrix ductility and fiber brittleness contribute to damage evolution. Overall, the images confirm that interfacial quality and fiber dispersion play a decisive role in determining the composite’s post-failure behavior.

These findings emphasize the need for enhanced fiber-matrix adhesion, potentially through surface modification or coupling agents, and improved processing techniques to ensure uniform filler distribution and superior mechanical performance under flexural loads.

The mechanical behavior and fracture resistance of leather–epoxy composites are predominantly governed by synergistic interactions between the fiber-matrix interface and associated toughening mechanisms. At optimal leather fiber volume fractions (~ 0.3), composites demonstrated enhanced flexural strength and quasi-ductile behavior, attributed to efficient stress transfer and activation of key energy-dissipating mechanisms^37^. The calculated interfacial shear stress of 48.75 MPa and critical fiber length of 1.54 µm reflect favorable stress distribution at the microscale, facilitating crack bridging and fiber pull-out during flexural loading^38^. These mechanisms act to delay crack propagation and increase the composite’s work-of-fracture, as evidenced by improved flexural strength (100.8 MPa) and fracture energy (13.5 J).

Microscopic examination via SEM confirmed the presence of prominent toughening mechanisms, namely fiber pull-out, interfacial debonding, and crack deflection. Fiber pull-out was frequently observed in the fractured regions, often accompanied by resin tearing and interfacial gaps measuring 2–5 µm. This phenomenon indicates frictional sliding between the fiber and matrix, leading to progressive failure and increased energy absorption. The sliding distances of 20–60 µm for pulled-out fibers support their significant role in mitigating abrupt fracture. Interfacial debonding was also visible at multiple fracture sites, contributing to stress redistribution and delaying matrix cracking. Additionally, the collagen-rich microstructure of leather fibers was found to induce crack deflection, as cracks were frequently observed to deviate at fiber-matrix boundaries, increasing fracture surface area and resistance to crack propagation.

The optical microscopy and SEM results revealed dominant micro-damage mechanisms, including fiber pull-out (20–60 μm in length), matrix cracking, and interfacial debonding. While these observations were primarily qualitative, the consistent occurrence of these features across multiple regions suggests their central role in energy dissipation and progressive failure. Due to the scope of this study and limitations in imaging resolution and sample preparation, a fully quantitative assessment of porosity or interfacial area ratios was not conducted. Future investigations will incorporate image-based morphometric analyses to statistically quantify porosity, particle dispersion, and bonding area coverage to better correlate microstructure with mechanical performance.

These micro-damage mechanisms-particularly crack deflection and fiber pull-out-act as effective toughening strategies by dissipating energy during crack propagation, thereby enhancing both fracture energy and strain-at-break. Their combined effect transitions the failure behavior from brittle to quasi-ductile, aligning with the observed increase in energy absorption capacity under flexural loading.

Quantitative damage analysis further supports the activation of these mechanisms. The dominant fracture modes included interfacial debonding (45%), cohesive matrix fracture (35%), and fiber rupture (20%). These values are consistent with previous studies on natural fiber composites, which identify interfacial phenomena as the primary contributors to improved energy dissipation under flexural stress.

Optical microscopy, including both bright and dark field techniques, revealed resin-starved zones, fiber misalignment, and microcracks in overfilled composites (> 0.4 fiber volume). These morphological features correlate with reductions in mechanical properties, as excess fibers tend to agglomerate, creating voids and reducing matrix continuity. This leads to premature failure due to localized stress concentrations. In contrast, fiber volume fractions below 0.2 resulted in brittle fracture modes dominated by matrix cracking, indicating insufficient fiber reinforcement.

The observed trends align with the predictions of shear-lag theory, which posits that there exist an optimal fiber length and volume for efficient load transfer. The experimental results validate this: while the introduction of leather fibers significantly enhanced toughness, performance plateaued or declined when fiber content exceeded dispersion capacity. Thus, the relationship between microstructural morphology and mechanical behavior is nonlinear, and optimal performance is achieved only under balanced interfacial conditions.

The findings of the present study can be better contextualized by considering recent research that explores the role of bio-based fillers and agro-waste reinforcements in epoxy composites. For instance, the study by Ramesh et al.^39^ examined the influence of wood dust fillers on the mechanical, thermal, water absorption, and biodegradation characteristics of jute fiber epoxy composites, demonstrating how lignocellulosic additives can significantly enhance material performance while promoting environmental sustainability. Similarly, Rajeshkumar et al.^40^ investigated fully bio-based agro-waste soy stem fiber-reinforced epoxy composites, emphasizing the role of surface modification techniques in improving interfacial adhesion and mechanical integrity. These studies highlight the relevance of waste-derived reinforcements and interface engineering concepts that directly align with the objectives of the present work. By incorporating leather waste into the epoxy matrix, the current study contributes to this evolving research domain, offering further evidence of how non-conventional bio-fillers can support the development of lightweight, sustainable composite materials suitable for semi-structural applications.

The integration of leather waste as reinforcement in epoxy composites is validated by both experimental and microscopic evidence. The toughening mechanisms identified-particularly fiber pull-out, interfacial debonding, and crack deflection-are essential in enhancing energy absorption and delaying catastrophic failure. These findings are consistent with literature on natural fiber-reinforced composites and provide further support for using agro-waste and bio-fillers in semi-structural composite applications. Future work may explore surface treatment of leather fibers to improve wettability and dispersion, thereby further enhancing the mechanical properties and sustainability of such bio-composites.

**Fig. 14:**
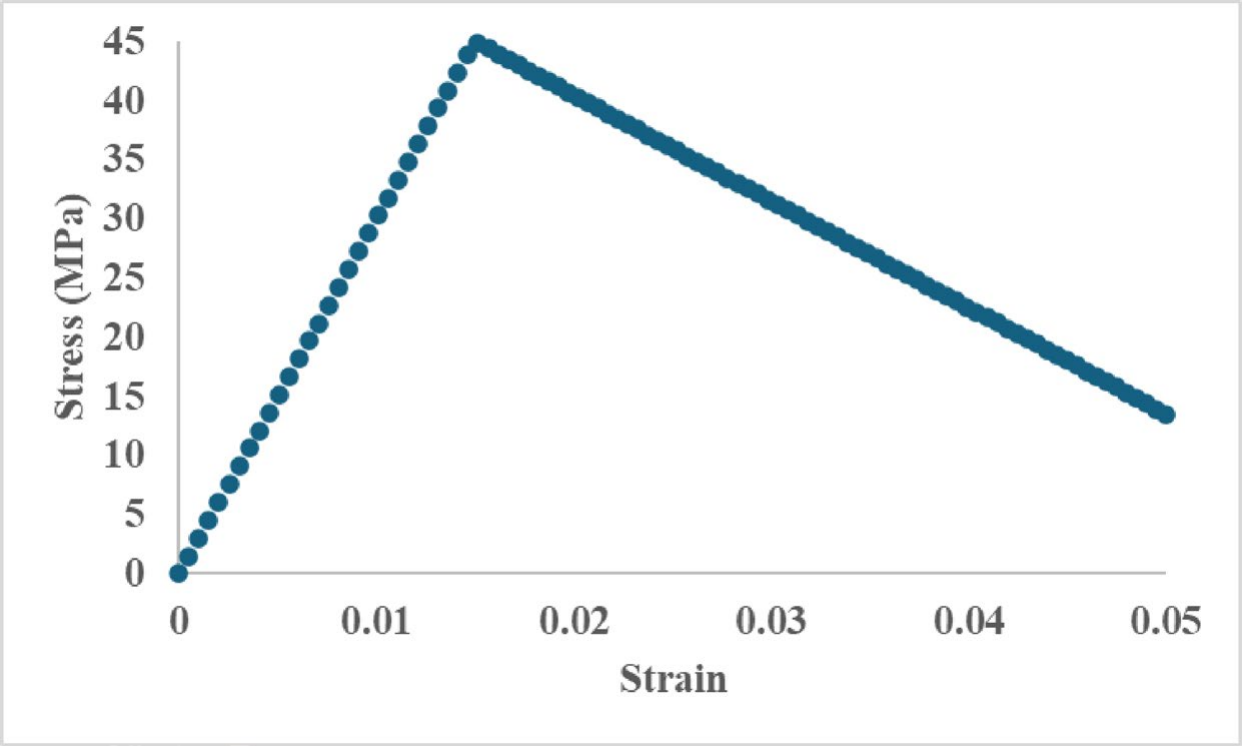
Predictive stress–strain curve for leather epoxy composite modeled using a bilinear viscoelastic-softening approach. The curve illustrates initial linear elastic behavior followed by post-yield softening due to interfacial debonding and matrix damage, reflecting typical failure mechanisms observed in experimental characterization.

**Fig. 15:**
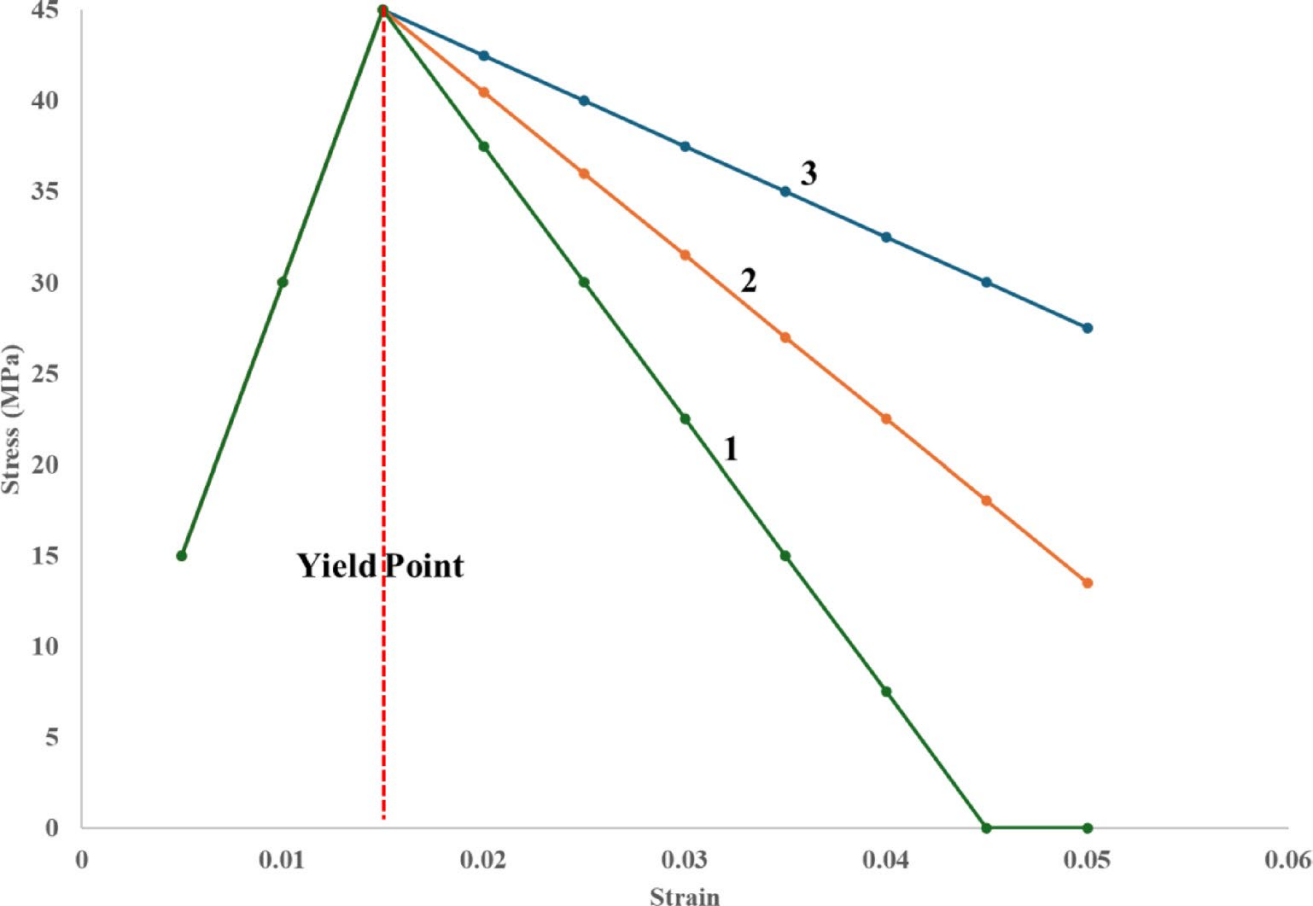
Parametric stress strain behavior with varying interfacial strength 1- strong interface, 2-Moderate interface, 3-Weak Interface.

Figures 14 and 15 illustrate a simplified bilinear viscoelastic-softening model developed to qualitatively simulate the characteristic stress–strain response observed in leather-epoxy composites subjected to three-point bending. As shown in Fig. 14, the model employs experimentally derived parameters, including an initial elastic modulus of approximately 3 GPa, a yield stress of ~ 45 MPa occurring at ~ 1.5% strain, and a descending post-yield slope to represent progressive stiffness degradation. This behavior is consistent with the onset of matrix microcracking, fiber-matrix interfacial debonding, and inelastic fiber pull-out. The model was implemented in MATLAB using piecewise linear constitutive relations and is intended as a first-level approximation to visualize composite deformation trends rather than serve as a predictive tool.

In Fig. 15, a parametric analysis is performed by varying the softening slope in the post-yield region to reflect different levels of interfacial adhesion-weak, moderate, and strong. Composites modeled with weaker interfaces show a sharp decline in stress post-yield, capturing brittle matrix failure and extensive fiber pull-out, as corroborated by SEM observations of voids and poor wetting. In contrast, the smoother softening behavior associated with stronger interfaces indicates better stress redistribution and damage tolerance due to improved fiber-matrix bonding and effective load transfer, consistent with observed microstructural features such as reduced interfacial gaps and enhanced resin infiltration. Although the model does not account for rate dependency or nonlinear viscoelasticity, its output closely parallels the empirical fracture patterns noted in microscopy and mechanical analysis. Thus, the model serves to underscore the influence of interfacial characteristics on composite toughness and offers a visual framework for guiding future optimization strategies in composite formulation.

Table 3 provides a comparative overview of the mechanical properties of the leather-epoxy composite developed in this study, alongside selected natural and synthetic fiber-reinforced epoxy systems^41,42^. The leather-epoxy composite exhibited a flexural strength of 100.8 MPa and a flexural modulus of 18.64 GPa, which surpasses several commonly used natural fibers such as jute (60–90 MPa; 80–12 GPa), flax (80–100 MPa; 10–15 GPa), and kenaf (70–95 MPa; 9–13 GPa). Although its absolute properties remain slightly below those of conventional glass fiber-epoxy composites (120–160 MPa; 20–30 GPa), the leather-epoxy system offers a substantially lower density (1.1 g/cm^3^) compared to glass fiber composites (~ 2.0 g/cm^3^), thereby delivering improved specific strength and stiffness. This advantage enhances its suitability for weight-sensitive applications^43,44^.

**Table 3:** Comparison of mechanical properties of leather epoxy composite with conventional natural and synthetic fiber composites.

	Composite type	Flexural strength (MPa)	Flexural modulus (GPa)	Density (g/cm^3^)
1	Leather-epoxy (This study)	100.8	18.64	1.1
2	Jute-epoxy	60–90	8–12	1.3
3	Flax-epoxy	80–100	10–15	1.4
4	Kenaf-epoxy	70–95	9–13	1.35
5	Hemp-epoxy	65–85	7–11	1.3
6	Glass fiber-epoxy	120–250	20–30	2.0

To further contextualize, literature reports on jute–epoxy composites at comparable fiber volume fractions (V _f_ ≈ 0.3) indicate flexural strengths in the range of 95–105 MPa and moduli between 12 and 16 GPa [Ramesh et al.^39^]. Similarly, flax-epoxy composites have achieved flexural strengths of ~ 110 MPa and moduli around 14 GPa [Rajeshkumar et al.^40^]. The current leather–epoxy composite thus lies at the upper end of jute systems and exceeds many flax and kenaf composites in stiffness. This superior modulus may be attributed to the inherent rigidity of leather fibers, the enhanced filler–matrix interlocking, and improved interfacial bonding, as evidenced by minimal fiber pull-out and uniform dispersion observed in SEM micrographs (Figs. 7, 8, 9). The observed failure mechanisms-fiber-matrix debonding, crack deflection, and localized matrix plasticization-correspond to the stress distribution patterns inferred from flexural stress–strain profiles.

The leather-epoxy system offers a promising balance between mechanical performance, sustainability, and lightweight characteristics, suggesting its viability as a structural alternative to traditional natural fiber composites in semi-structural applications. While the current study establishes a foundational understanding, future work should include larger sample sizes, long-term durability testing, and environmental exposure assessments to confirm its practical applicability.

Leather waste, when used as a reinforcement in polymer composites, presents significant potential due to its unique structural properties and environmental value. Unlike traditional biofibers such as jute or flax, leather particles exhibit high density, toughness, and the ability to resist crack propagation, which contribute positively to the composite’s flexural performance. Additionally, repurposing leather waste addresses major sustainability challenges by diverting industrial byproducts from landfills and reducing the environmental footprint associated with virgin fiber processing. While plant-based fibers may perform better in tensile applications, leather reinforcement offers a favorable combination of moderate mechanical strength, energy absorption, and ecological benefit-especially in applications where particulate fillers are suitable, such as panels, automotive interiors, and construction elements. This study demonstrates the preliminary promise of such composites, but further validation is needed across broader loading conditions and long-term use scenarios.

## Conclusion

This study examined the mechanical performance and failure mechanisms of leather particle-reinforced epoxy composites under quasi-static three-point bending. At a fiber volume fraction of approximately 0.3, the composites achieved a flexural strength of 100.8 MPa and a modulus of 18.64 GPa, with a low coefficient of variation (2.95%), indicating consistent laboratory-scale reproducibility. These values are comparable to those reported for conventional natural fiber composites such as jute, flax, and kenaf, indicating that processed leather waste can function as a competitive reinforcement under similar test conditions. Fractographic observations from optical microscopy and SEM revealed characteristic damage and toughening mechanisms, including fiber pull-out, interfacial debonding, matrix cracking, and void-assisted crack propagation. The introduction of leather fibers increased the strain-at-break by approximately 40% and raised the fracture energy from 8.2 J (neat epoxy) to 13.5 J, indicating a transition from brittle failure to more energy-absorbing, quasi-ductile behavior. The findings reported here are limited to a single fiber volume fraction (V_f_ ≈ 0.3), and broader optimization will require systematic studies across multiple V_f_ values (0.1–0.4) and alternative processing routes before general conclusions about material performance can be drawn.

Although the V_f_ = 0.3 formulation displayed promising flexural and fracture responses, additional studies across a wider span of fiber volume fractions are needed to determine optimal reinforcement levels and to assess how property trends evolve with scale-up. Processing-related anomalies such as resin-rich regions, fiber agglomeration, and micro-voids also highlight the importance of refining infusion and dispersion protocols. Future work should investigate surface treatments, coupling agents, and potential hybridization with micro- or nano-fillers to strengthen fiber–matrix interfacial bonding. Furthermore, long-term performance cannot be inferred from the present quasi-static tests alone. Comprehensive durability assessments including fatigue, creep, moisture uptake, and thermal ageing are necessary to evaluate the material’s stability under service conditions. Complementary predictive approaches, such as finite element modelling and data-driven optimization frameworks, may also support systematic material development. This exploratory study contributes to the understanding of micromechanical toughening mechanisms in recycled leather-epoxy composites and demonstrates that post-consumer leather fibers exhibit promising behavior for semi-structural or moderately loaded applications. Nevertheless, their potential use in domains such as automotive interiors, construction panels, or impact-mitigating systems requires further verification through comprehensive durability assessments, process optimization, and multi-scale modelling.

## Data Availability

Data sets generated during the current study are available from the corresponding author on reasonable request.

## Code availability

The authors confirm that the data supporting the findings of this study are available within the article.
